# Experiment to Demonstrate Pesticide-Induced Antimicrobial Resistance (AMR): An Emerging Health Threat

**DOI:** 10.7759/cureus.54243

**Published:** 2024-02-15

**Authors:** Rahil Pasha S A, Namitha B N, Warisha Ismail, Repati Gowri, Arvind Natarajan

**Affiliations:** 1 Microbiology, Sri Devaraj Urs Academy of Higher Education and Research, Kolar, IND

**Keywords:** imidacloprid, hazardous substances, pesticide induced antimicrobial resistance, antimicrobial resistance, pesticide

## Abstract

Background

Pesticides, including insecticides, herbicides, and fungicides, are essential for global food production, boosting crop yields, and preventing disease transmission. However, their excessive and improper use raises concerns about potential long-term consequences, affecting microbial ecosystems and fostering antimicrobial resistance.

Materials and methods

The objective of the study was to identify the effect of the pesticide compound (Imidacloprid 17.1% w/w) on the ATCC *Escherichia coli*. An experiment was conducted on ATCC *Escherichia coli* 27852. A 0.5 McFarland suspension of the strain was incubated in the presence of a pesticide compound (Imidacloprid 17.1% w/w) at a dilution of 1:4, 1:8, and 1:16. at 370C. Antibiotic susceptibility for cefoxitin, ciprofloxacin, ceftazidime, amikacin, and imipenem was determined via the Kirby-Bauer disk diffusion test at intervals of 24 hours, 48 hours, seven days, and 21 days. The results were then compared to the standard zone of inhibition diameter for ATCC *Escherichia coli* 27852 by Clinical and Laboratory Standards Institute (CLSI) guidelines.

Results

No bacterial growth was detected at pesticide dilutions of 1:1 and 1:2, indicating their inability to tolerate high pesticide concentrations. However, growth became evident at a 1:4 dilution and beyond, with mutants thriving within the inhibition zone. The experiment caused significant alterations in the inhibition zone sizes for all antibiotics, especially notable with imipenem, amikacin, and ceftazidime compared to the initial zone size for ATCC *Escherichia coli *27852.

Conclusion

Our study concludes that the pesticide (Imidacloprid 17.1% w/w) significantly influences antibiotic resistance, especially with carbapenems, aminoglycosides, and cephalosporins in the tested groups at various concentrations and durations of exposure.

## Introduction

The World Health Organization (WHO) has classified families of pesticides as “hazardous substances” [1,2]. Pesticides were considered defenders of global food security because they were designed to disrupt vital processes in pests, like nerve signaling, metabolism, and cell functions. However, recent research has revealed that pesticides can harm human health and non-targeted organisms by altering physiological mechanisms that aren't exclusive to their target pests [3,4]. The significant increase in the prevalence of cancer, leukemia, inflammatory bowel disease (IBD), and neurological disorders highlights the serious public health issue associated with prolonged human exposure to pesticides [5,6]. Efforts to curb pesticide use have fallen short due to the current agricultural focus on maximizing crop yields through the widespread use of pesticides [7].

The colonization of the human gut begins at birth and evolves continuously, influenced by environment and diet. [8]. The gut microbiota plays a vital role in digestive health by supporting intestinal cell regeneration, mucus production, nutrient absorption, and the fermentation of indigestible substances [9]. The gut microbiota can be disrupted by exposure to substances like drugs, antibiotics, and, especially in this context, pesticide residues present in contaminated food and water. The current study illustrates how normal intestinal flora is impacted by pesticide residues in the development of antibiotic resistance.

## Materials and methods

The selection of pesticides and bacterial strain

This study was conducted at Sri Devaraj Urs Academy of Higher Education and Research, Kolar, India. We arbitrarily selected the pesticide Imidacloprid (17.1% w/w), which is used frequently in the local area. ATCC *Escherichia coli* 27852 was chosen for this study because *Escherichia coli* is the predominant aerobic microbiota in the human gut and is widely used as a susceptible and negative control in many relevant studies [10,11].

Stock ATCC *E. coli* was inoculated onto a nutrient agar plate. Following 20 hours of incubation, fresh colonies were taken and inoculated in nutrient broth, and a 0.5 McFarland suspension of the strain was prepared and employed as the starting inoculum for the evolutionary experiments.

The pesticide compound (Imidacloprid 17.1% w/w) was diluted using a double dilution method (1:2, 1:4, 1:8). The dilutions were carried out with the anticipation that the bacteria might not withstand the elevated concentration of the pesticide compound.

A 0.5 McFarland suspension of ATCC *Escherichia coli* 27852 in nutrient broth was mixed with pesticide compound (Imidacloprid 17.1% w/w) at a dilution of 1:2, 1:4, 1:8, and 1:16, and the tubes were incubated at 37^0^C. The mixture was inoculated onto nutrient agar, and antibiotic susceptibility for cefoxitin, ciprofloxacin, ceftazidime, amikacin, and imipenem was determined using the Kirby-Bauer disk diffusion test at the end of 24 hours, 48 hours, seven days, and 21 days, and the results were compared with the standard zone of inhibition of diameter of ATCC *Escherichia coli* 27852 as per the Clinical and Laboratory Standards Institute (CLSI) guidelines. Additionally, control groups were used for comparison, consisting of two independent populations not exposed to any pesticide.

## Results

Trajectories of phenotypic resistance in *E. coli* populations with and without pesticide co-stressors

No bacterial growth was observed when the bacteria were incubated with the pesticide compound at dilutions of 1:1 and 1:2, signifying their inability to endure high pesticide concentrations. Nonetheless, bacterial growth became apparent from a 1:4 dilution and beyond. Interestingly, mutants were found to flourish within the zone of inhibition. The experiment resulted in modifications to the diameter of inhibition zones for all antibiotics, with the most significant alterations observed in the cases of imipenem, amikacin, and ceftazidime when compared to the initial inhibition zone diameter for ATCC *Escherichia coli* 27852.

According to the data presented in Table 1, a notable decrease in the zone diameter was observed for imipenem at a 1:4 concentration after 48 hours. Furthermore, it was evident that even at the 1:16 concentration, a substantial reduction in the inhibition zone was noted. With amikacin, a significant decrease in the inhibition zone diameter was noted at the 1:4 concentration after seven and 21 days of incubation, and a significant difference was also seen at the 1:8 concentration. Still, no notable change was observed at the 1:16 concentration. Regarding ceftazidime, a noteworthy decrease in the zone diameter was observed at the 1:4 concentration after seven and 21 days. However, no significant alterations were noted at other concentrations in subsequent periods.

**Table 1:** Zone of inhibition of ATCC Escherichia coli in the presence of pesticide compounds at different concentrations

Antimicrobial agent	Disk strength	Quality control limit	Zone of inhibition without pesticide	Dilution of pesticide compound	Zone of inhibition with pesticide at 24hrs	Zone of inhibition with pesticide at 48hrs	Zone of inhibition with pesticide at seven days	Zone of inhibition with pesticide at 21 days
Cefoxitin	30 mcg	23-29 mm	28	1:4	23	25	24	24
				1:8	25	24	23	23
				1:16	24	24	28	28
Ciprofloxacin	5 mcg	29-38 mm	36	1:4	39	30	34	34
				1:8	29	39	29	29
				1:16	35	34	36	36
Ceftazidime	30 mcg	25-32 mm	26	1:4	26	28	23	23
				1:8	25	29	27	27
				1:16	25	30	28	28
Imipenem	10 mcg	26-32 mm	28	1:4	23	16	23	23
				1:8	15	16	25	25
				1:16	25	19	24	24
Amikacin	30 mcg	19-26 mm	22	1:4	24	24	18	18
				1:8	23	22	18	18
				1:16	23	24	23	23

## Discussion

Antimicrobial resistance (AMR) represents a substantial global challenge to healthcare and development. The World Health Organization (WHO) has officially recognized AMR as one of humanity's top ten global public health issues. The main drivers behind the rise of drug-resistant pathogens stem from the improper and excessive use of antimicrobial agents. An emphasis on optimizing crop production by extensively employing pesticides has resulted in the emergence of pesticide-induced antimicrobial resistance within the human gut microbiota.

The experiment induced variations in the zone of diameter of inhibitions in all antibiotics but was most significant in imipenem, amikacin, and ceftazidime compared with the quality control limits of ATCC *Escherichia coli* 27852. According to a study conducted by Kurenbach B, *E. coli*, and *Salmonella enterica serovar Typhimurium* were exposed to three herbicides: dicamba (Kamba500,® Nufarm, Otahuhu, New Zealand), 2,4-dichlorophenoxyacetic acid (2,4-D), and glyphosate (Roundup,® Monsanto, Melbourne, Australia), and they displayed different responses to antibiotics. In the case of *S. enterica serovar Typhimurium*, exposure to Kamba® and 2,4-D resulted in increased tolerance to ampicillin, chloramphenicol, ciprofloxacin, and tetracycline. Similarly, *E. coli* exhibited a comparable response, except for ampicillin tolerance not being increased. Additionally, for both species, exposure to Roundup® led to an elevated tolerance to ciprofloxacin and kanamycin.” [12].

Yue Xing's study exposed *E. coli* K-12 to environmentally relevant pesticide levels and streptomycin for 500 generations, resulting in a remarkable, more than 15-fold increase in streptomycin resistance due to substantial changes in phenotypic, genotypic, and fitness traits [13].

Exposing organisms to pesticides can result in genetic changes that contribute to antibiotic resistance. Resistant mutants exposed to pesticides displayed genetic alterations that likely affect gene expression, biofilm formation, and defense against oxidative stress when exposed to antibiotics. This phenomenon illuminates the intricate interaction between pesticides and antibiotics, suggesting pesticide exposure may exacerbate antibiotic resistance in the gut flora.

Limitations of the study

The research could have encompassed gram-positive and gram-negative bacteria, utilizing the minimum inhibitory concentration (MIC) method. Furthermore, the study might have incorporated molecular techniques to validate genetic changes within the bacterial strains and detect resistance genes, thereby identifying the underlying resistance mechanisms.

## Conclusions

Our study reveals that Imidacloprid 17.1% w/w significantly influences antibiotic resistance, especially with carbapenems, aminoglycosides, and cephalosporins in the tested groups at various concentrations and durations of exposure.
